# Linked Mutations at Adjacent Nucleotides Have Shaped Human Population Differentiation and Protein Evolution

**DOI:** 10.1093/gbe/evz014

**Published:** 2019-01-23

**Authors:** James G D Prendergast, Carys Pugh, Sarah E Harris, David A Hume, Ian J Deary, Allan Beveridge

**Affiliations:** 1 The Roslin Institute, The University of Edinburgh, Midlothian, United Kingdom; 2 Centre for Cognitive Ageing and Cognitive Epidemiology, Department of Psychology, The University of Edinburgh, United Kingdom; 3 Centre for Genomic and Experimental Medicine, MRC Institute of Genetics and Molecular Medicine, The University of Edinburgh, United Kingdom; 4 Mater Research Institute-University of Queensland, Woolloongabba, Queensland, Australia; 5 Glasgow Polyomics, College of Medical, Veterinary and Life Science, University of Glasgow, United Kingdom

**Keywords:** sequential dinucleotide mutations, multinucleotide polymorphisms, multinucleotide mutations, human mutation, DNA repair, epistatic selection, SDM, MNP

## Abstract

Despite the fundamental importance of single nucleotide polymorphisms (SNPs) to human evolution, there are still large gaps in our understanding of the forces that shape their distribution across the genome. SNPs have been shown to not be distributed evenly, with directly adjacent SNPs found unusually frequently. Why this is the case is unclear. We illustrate how neighboring SNPs that cannot be explained by a single mutation event (that we term here sequential dinucleotide mutations [SDMs]) are driven by distinct processes to SNPs and multinucleotide polymorphisms (MNPs). By studying variation across populations, including a novel cohort of 1,358 Scottish genomes, we show that, SDMs are over twice as common as MNPs and like SNPs display distinct mutational spectra across populations. These biases are not only different to those observed among SNPs and MNPs but are also more divergent between human population groups. We show that the changes that make up SDMs are not independent and identify a distinct mutational profile, CA → CG → TG, that is observed an order of magnitude more often than expected from background SNP rates and the numbers of other SDMs involving the gain and deamination of CpG sites. Intriguingly particular pathways through the amino acid code appear to have been favored relative to that expected from intergenic SDM rates and the occurrences of coding SNPs, and in particular those that lead to the creation of single codon amino acids. We finally present evidence that epistatic selection has potentially disfavored sequential nonsynonymous changes in the human genome.

## Introduction

Single nucleotide polymorphisms (SNPs) are the pillars of modern genetics studies (Brookes 1999; Gray et al. 2000). From their use in genome-wide association studies to map the genetics of diseases (Bush and Moore 2012), to studying patterns of evolution (Morin et al. 2004), SNPs are widely used and studied across fields. A common implicit assumption across these studies is that SNPs are independent (Schrider et al. 2011), with each substitution assumed to have resulted from a distinct mutational event. However, the distribution of SNPs across the genome has been known for some time to not be even, with not only polymorphisms (Amos 2010; Hodgkinson and Eyre-Walker 2010) but also fixed differences between species clustering in the genome (Bazykin et al. 2004). Where multiple base substitutions are found in the same genomic region, the derived alleles are more often found on the same haplotype (Schrider et al. 2011), suggesting that the two changes have not occurred independently.

Directly neighboring polymorphisms are particularly enriched in the human genome (Hodgkinson and Eyre-Walker 2010), though why this is the case is not fully understood. Previous studies of trios have shown that many of these changes have arisen in a single generation as multinucleotide polymorphisms (MNPs) (Schrider et al. 2011; Besenbacher et al. 2016), which are particularly enriched with GA → TT and GC → AA changes, suggesting that they are linked to error prone replication by polymerase zeta (Harris and Nielsen 2014). However, not all sites of neighboring changes can be explained by a single mutational event. Many neighboring polymorphisms occur at different allele frequencies in the population suggesting that they have arisen from two distinct mutations (Hodgkinson and Eyre-Walker 2010), which we term here sequential dinucleotide mutations (SDMs). These clustered changes have been comparatively understudied and may simply reflect mutational hotspots but may also be the result of selection, with the impact of an initial deleterious SNP being at least partly corrected by a second neighboring change (Davis et al. 2009). One obvious circumstance is where the impact of a deleterious nonsynonymous change is offset by a second polymorphism nearby. For example, nonsynonymous changes are less often found on highly expressed haplotypes, suggesting that selection has favored particular combinations of coding and regulatory alleles (Lappalainen et al. 2011). Likewise there is evidence that selection has favored particular combinations of nonsynonymous changes spanning different amino acids in the same protein (Breen et al. 2012).

In this study, we therefore focused on SDMs comprising two neighboring changes that cannot be readily explained by a single mutational event and investigate whether they simply reflect two independent but neighboring polymorphisms or whether the second change depends on the first. The human mutation spectrum has diverged between human populations, with particular SNPs in particular sequence contexts more common in different continental groups (Harris and Pritchard 2017). Accordingly, we also explore whether SDM fractions have diverged between population groups and whether any divergence simply mirrors differences in SNP or MNP mutational profiles. We also characterize the functional importance of these SDMs by studying the selective pressures acting upon them and whether they have favored particular pathways through the amino acid code.

## Materials and Methods

### Variant Calling

**Fig. 1.**
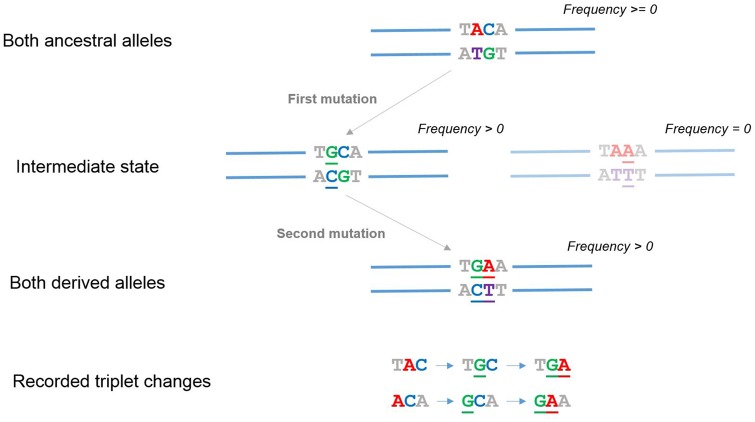
—Defining the SDMs studied in this analysis. In this study, we focused on SDMs involving neighboring nucleotides that could not be readily explained by a single mutational event. Haplotypes containing just one of the two changes that make up the SDM had to be observed among the individuals (we assumed that reverting mutations and recombination events between neighboring bases were rare). Using information on which alleles were ancestral, we then inferred the order of changes, with each SDM recorded twice, according to their immediate 5′ and 3′ nucleotides. This allowed downstream analysis of their impact on codons and comparisons to SNPs in the same triplet contexts.

1000 Genomes Consortium version 3 phased haplotypes along with information on their ancestral alleles were obtained from http://ftp.1000genomes.ebi.ac.uk/vol1/ftp/release/20130502/; last accessed February 14, 2019. After excluding variants with missing or low confidence ancestral allele annotations, neighboring variants where both derived alleles were observed together on the same haplotype were flagged as potential SDMs. As the focus of this analysis was on SDMs originating from two neighboring mutation events, only SDMs where haplotypes also existed (in any population) which carried the derived allele at one but not the other variant were kept, that is, the two changes were observed at different allele frequencies. See figure 1 for more details on how SDMs were defined in this study. This led to 169,702 putative MNPs being excluded where only two haplotypes were observed. Due to the very low probability of recombination events occurring between neighboring bases in the human genome, we also excluded SDMs where both combinations of one derived and one ancestral allele were observed. Following this filtering 377,766 neighboring pairs of polymorphisms remained.

As switch errors would impact the ability to correctly call SDMs, we assessed the proportion of incorrectly phased alleles in 26 randomly chosen individuals (one from each subpopulations) using GATK’s ReadBackedPhasing tool, which uses the co-occurrence of alleles in the same read to infer phase at nearby variants. On average only 0.34% of SDMs were phased discordantly to the original data using this approach (a further 1.7% could not be phased using the read backed phasing approach alone). This number was found to be relatively consistent across population groups (0.28% in Europeans to 0.4% in Africans).

Illumina HiSeq X paired-end sequencing data for 1,370 Lothian Birth Cohort (Deary et al. 2012; Taylor et al. 2018) individuals (mean sequencing depth of 36×) were aligned to the build 38 version of the human reference genome using BWA (Li and Durbin 2009). Variants were called using GATK (DePristo et al. 2011) according to its recommended best practices. This included the use of GATKs HaplotypeCaller software that implements read based phasing of nearby alleles. After checking identities with previous array data, excluding duplicate individuals and those displaying excessive levels of heterozygosity 1,358 individuals remained. All SNP coordinates were then lifted over to build 37 so as to match the 1000 genomes data set using Crossmap (Zhao et al. 2014). Ancestral sequences available at http://ftp.1000genomes.ebi.ac.uk/vol1/ftp/phase1/analysis_results/supporting/ancestral_alignments/; last accessed February 14, 2019. were used to determine the ancestral alleles of each SNP, and as with the 1000 genomes data only those variants with high-confidence calls were kept (see Paten et al. [2008] for more details). As with the 1000 genomes ancestral sequences, these are derived from a six-way alignment of primate genomes with only sites where the ancestral allele is consistent across multiple further sequences (not including the human sequence in question) being annotated as high confidence. Eighty-six percent of ancestral allele calls matched three or more other sequences in the alignment. SDMs were subsequently called using the same approach as the 1000 genomes data.

All SNPs were annotated using variant effect predictor (McLaren et al. 2016) with gene models from the version 85 release of Ensembl. SDMs were subsequently reannotated using custom python scripts to correctly annotate the consequence of the double base change in coding regions.

All noncoding SNPs, MNPs, and SDMs were defined with respect to their triplet context on the reference strand. This meant that all noncoding SDMs were recorded twice, once with respect to their immediate 5′ neighbor and once with respect to their 3′ neighbor (fig. 1). Noncoding SNPs were recorded with respect to all three frames within which they fell. On the other hand, coding polymorphisms were recorded with respect to just the actual codon within which they occurred. This enabled the direct comparison of the occurrence of SNPs and SDMs inside annotated codons to matching triplet bases outside coding regions.

### Calculating Observed and Expected Numbers of SDMs

The relative occurrence of particular mutation types was calculated as in previous studies of SNPs (Harris and Pritchard 2017). First, the number of distinct changes in a particular triplet context (*m*) was counted in each population (P). Then this count, *C*_p_(*m*) was converted to a mutational fraction by dividing it by the count of all observed changes. For SDMs, an individual was only a carrier if at least one of their haplotypes carried both derived alleles at the corresponding pair of nucleotides.

In various analyses, the two changes that comprise an SDM were separated into those that came first (i.e., the derived allele at just one of the two nucleotides is observed by itself in the population, see fig. 1) and those that came second. Their relative occurrences were also calculated as above.

The expected SDM frequencies of occurrence were derived from background SNP numbers by calculating the conditional probability of observing an SDM comprising the corresponding two changes (eq. 1).

(1) P(Change1 then Change2) = P(Change1)·P(Change2|Change1),

where P(Change1) is the proportion of SNPs displaying the corresponding change when defined by their triplet context. P(Change2|Change1) is the proportion of SNPs displaying the same change as Change 2 among all SNPs with the same ancestral triplet and where the change is at a position in the triplet neighboring the location of the first change. For example, if the first change was AAA > ATA and the second change ATA > ATC, P(Change2|Change1) would correspond to the number of ATA > ATC intergenic SNPs divided by the sum of all ATA > ATB and ATA > BTA changes (where B can be any nucleotide except A). This therefore accounts for the fact that the second change in an SDM had to occur at a base neighboring, but not at, the location of the first, and involve the triplet the first change had created.

To calculate the expected number of coding SDMs given their frequency of occurrence in intergenic regions, we first corrected the counts of each intergenic SDM for the difference in triplet occurrence between coding and intergenic regions. This was done for each SDM by dividing their observed number in intergenic regions by the ratio of intergenic to coding triplet counts for the corresponding ancestral triplet. To account for the general underrepresentation of SDMs in coding regions, we then divided this value for each SDM by the sum across all SDMs to get the relative, normalized mutational fractions of each change. This was finally multiplied by the total number of all coding SDMs so that the observed and expected counts were on the same scale.

### Statistical Analyses

To test whether the impact of CpG dynamics on SDM numbers depended on the form of the original base change, we fit the negative binomial generalized linear model specified in equation (2).

(2) $${\text{obsSDMCount}}_{i}=µ+{\text{ expSDMCount}}_{i}\beta_{l}+{\text{ firstBaseChange}}_{i}\beta_{m}+{\text{ cpgChange}}_{i}\beta_{n}+{\text{ firstBaseChange}}_{i}{\text{cpgChange}}_{i}\beta_{\mathrm{mn}}+{ɛ}_{i},$$

where obsSDMCount_*i*_ corresponds to the observed number of SDMs of type *i* (e.g., AAA > ATA > ATT), expSDMCount_*i*_ is the expected number given background SNP mutational fractions (see eq. 1) and firstBaseChange_*i*_ and cpgChange_*i*_ are the first base change and impact of both changes on any CpG sites, respectively. The significance of the interaction term was assessed using analysis of variance (ANOVA).

Comparisons of the relative mutational fractions of different forms of changes was carried out as in Harris and Pritchard (2017), that is, we used their iterative approach of undertaking conditionally independent chi-square tests to try and minimize false positive significant results. To test for the enrichment of specific codon changes among coding SDMs, having accounted for background rates of coding SNP and intergenic SDM changes, we used multiple linear regression as specified in equations (3)–(6).

(3) $${\text{firstCodingSDMCount}}_{i}=µ+{\text{firstIntergSDMCount}}_{i}\beta_{l}+{\text{ratio}}_{i}\beta_{o}+{\text{funcClass}}_{i}\beta_{m}+{\text{cpgChange}}_{i}\beta_{n}+{\text{funcClass}}_{i}{\text{cpgChange}}_{i}\beta_{\mathrm{mn}}+{ɛ}_{i},$$

(4) $${\text{firstCodingSDMCount}}_{i}=µ+{\text{codingSNPCount}}_{i}\beta_{l}+{\text{funcClass}}_{i}\beta_{m}+{\text{cpgChange}}_{i}\beta_{n}+{\text{funcClass}}_{i}{\text{cpgChange}}_{i}\beta_{\mathrm{mn}}+{ɛ}_{i},$$

(5) $${\text{secondCodingSDMCount}}_{i}=µ+{\text{secondIntergSDMCount}}_{i}\beta_{l}+{\text{ratio}}_{i}\beta_{o}+{\text{funcClass}}_{i}\beta_{m}+{\text{cpgChange}}_{i}\beta_{n}+{\text{funcClass}}_{i}{\text{cpgChange}}_{i}\beta_{\mathrm{mn}}+{ɛ}_{i},$$

(6) $${\text{secondCodingSDMCount}}_{i}=\;µ+{\text{codingSNPCount}}_{i}\beta_{l}+{\text{funcClass}}_{i}\beta_{m}+{\text{cpgChange}}_{i}\beta_{n}{+\text{ funcClass}}_{i}{\text{cpgChange}}_{i}\beta_{\mathrm{mn}}+{ɛ}_{i},$$

where firstCodingSDMCount_*i*_ corresponds to the number of distinct first changes among coding SDMs that match change *i*, where each *i* is 1 of the 576 possible single base difference between 2 codons. secondCodingSDMCount_*i*_ is the corresponding count among the second changes of coding SDMs and firstIntergSDMCount_*i*_ and secondIntergSDMCount_*i*_ are the corresponding counts among the same nucleotide triplets at intergenic SDMs. codingSNPCount_*i*_ is the count of the same change observed among coding SNPs, funcClass_*i*_ is the functional impact of the corresponding change (missense, stop gained, etc.) and cpgChange_*i*_ is the impact on any CpG sites (lost or created). Ratio_*i*_ is the ratio of the counts of the corresponding ancestral triplet in intergenic and coding regions. Any interaction effect between the functional impact of the given codon change and its impact on CpG sites is represented by *β_mn_*. The results of these four models are shown in supplementary figures S16 and S17, Supplementary Material online. Multiple linear regression was also used in the test for epistatic selection among coding SDMs as specified in equation (7).

(7) $${\text{codingSDMCount}}_{i}=µ+{\text{ intergSDMCount}}_{i}\beta_{l}+{\text{ funcClass}2}_{i}\beta_{m}+{\text{ codonCount}}_{i}\beta_{n}+{\text{ cpgChange}1}_{i}\beta_{o}+{\text{ cpgChange}}_{i}\beta_{p}+{ɛ}_{i},$$

where codingSDMCount_*i*_ is the number of each SDM, *i*, in the genome where *i* is restricted to the 663 SDM where the first change is missense. intergSDMCount_*i*_ is the count of the same SDM, *i*, in intergenic regions. funcClass2_*i*_ is the functional impact of the second change in SDM *i*, and cpgChange1_*i*_ and cpgChange2_*i*_ are the impact on CpG sites of the first and second changes in the SDM, respectively. codonCount_*i*_ is the number of codons that encode the final amino acid created by the SDM, to account for the fact that amino acids with only one codon are generally favored by SDMs. A goodness-of-fit test confirmed that the Poisson model suitably fit the data (chi-square *P* = 0.997).

**Fig. 2.**
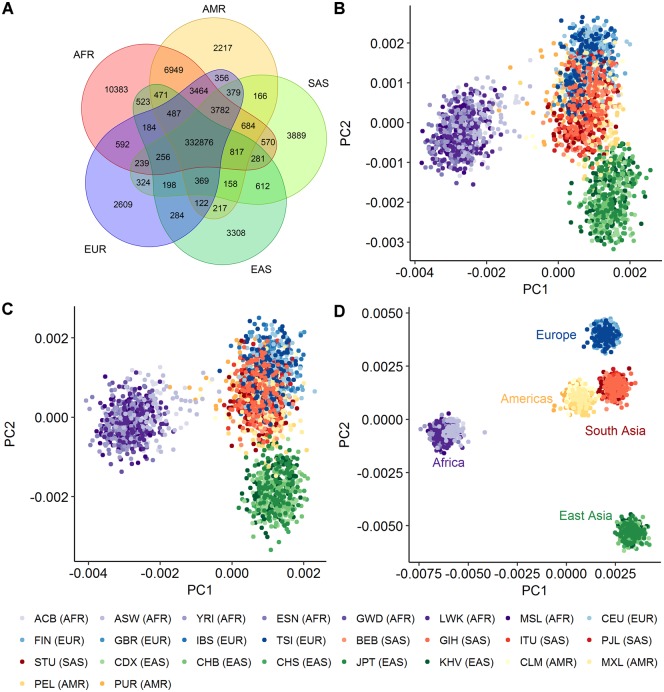
—Divergence in SDM mutational fractions between human populations. (*A*) Venn diagram of the number of intermediate haplotypes observed across different populations. (*B*) Principal component analysis of individuals according to the mutational fraction of each first change in the SDMs they carry, defined by their triplet context. Points are colored according to the continental group to which they belong and the corresponding 1000 Genomes Consortium three letter population codes are shown below. Although restricted to SNPs forming part of SDMs only, this plot largely mirrors that derived using all SNPs in the same populations presented in Harris and Pritchard (2017) at https://doi.org/10.7554/eLife.24284.006. (*C*) The same as (*A*) but for the second change in SDMs. (*D*) Principal component analysis of individuals according to the mutational fraction of the SDM changes they carry defined by triplet context.

## Results

To investigate the frequency of occurrence of neighboring mutations in different populations, we defined SDMs in the 1000 genomes phase 3 data set (1000 Genomes Project Consortium 2015) using the approach illustrated in figure 1. In this study, we focused specifically on changes across neighboring nucleotides that, due to being found at different allele frequencies, cannot be readily explained by a single mutational event. All of these SDMs were annotated with respect to their ancestral alleles, triplet nucleotide context and occurrence in each individual (see methods), allowing us to infer the order of nucleotide changes. SNPs were defined in the same way to enable direct comparisons of their mutational profiles to SDMs.

We observed that a typical individual carries over 14,000 of these compound SDMs (supplementary fig. S1, Supplementary Material online) of which on average 27 fall within a protein coding region. The distribution of SDMs across the genome broadly follows that of SNPs (supplementary figs. S2–S6, Supplementary Material online), with the exception of the major histocompatibility complex on chromosomes 6, that carries an unusually high proportion of SDMs despite its high SNP density (supplementary fig. S7, Supplementary Material online).

Examination across populations highlights that the intermediate haplotype is generally common (supplementary fig. S8, Supplementary Material online) and for 88% of SDMs, it is observed across all continental groups, suggesting that the first change for many SDMs occurred prior to the human migration out of Africa (fig. 2*A*).

**Fig. 3.**
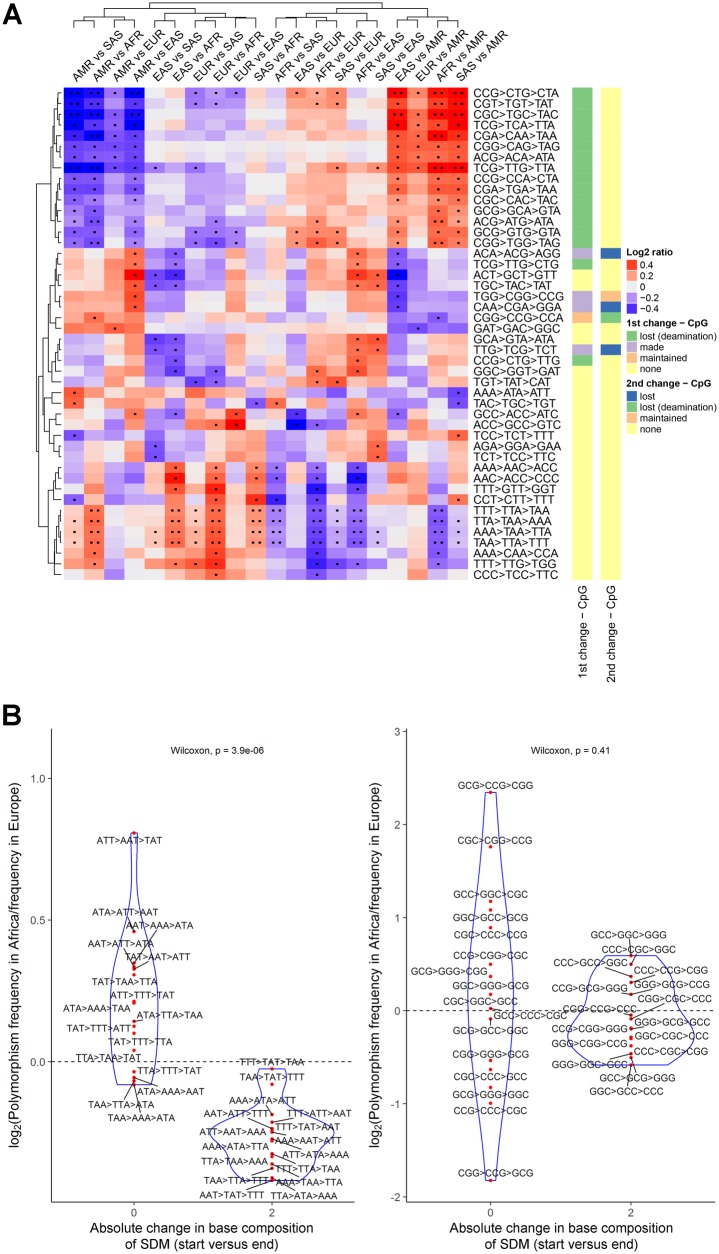
—SDMs that differ between population groups. (*A*) Log_2_ ratios of the frequencies with which selected SDMs occur in different continental groups. Only SDMs found at at least 150 sites in each population and with a Log_2_ ratio >= 0.3 and a *P* < 0.05 in at least one population comparison are shown in this plot. One dot indicates the corresponding comparison was associated with an uncorrected Fisher’s exact *P* < 0.05, two dots that the false discovery rate (*q* value) was <0.05. Red cells indicate the corresponding SDM is relatively enriched in the first named continental group, blue that the change is enriched in the second population. (*B*) The relative enrichment of selected SDMs in African versus European populations. (Left) SDMs that exclusively involve weak base pairs (adenine and thymine) broken down by their net impact on the base composition of each strand. (Right) SDMs that exclusively involve strong base pairs (guanine and cytosine).

### SDMs Show Biases between Human Populations Distinct to Those among SNPs

Despite being a reduced summary statistic compared with individual genotypes, comparisons of the numbers of SNPs found in different triplet contexts between individuals have been shown to separate out the major human population groups (Harris and Pritchard 2017). This has been attributed to differences in the large number of genes that control DNA mutation and repair between populations. Characterization of the individual SNPs that make up SDMs recapitulates the patterns observed in this previous study. Principal component analysis (PCA) of the frequency of occurrence of the first and second changes in compound SDMs, defined by their sequence context, shows highly similar patterns to that observed in Harris and Pritchard (2017) (fig. 2*B* and *C*). Despite their more limited numbers, the mutational profile of SDMs can though even more effectively separate out the major human subpopulations (fig. 2*D*); with certain types of SDMs in specific nucleotide contexts enriched in different populations. Surprisingly, unlike the PCAs of individual SNPs, the American continental group separates in this analysis, in part driven by a depletion for CG → TG → TA SDMs among these individuals (fig. 3*A*). In contrast, SDMs are relatively depleted at AT-rich triplets in African populations, and in particular SDMs where the mutations have the effect of switching AT base composition between strands (i.e., that involve multiple neighboring A:T → T:A or T:A → A:T mutations; fig. 3*B*). To ensure these results were not due to ancestral allele misidentification, we repeated the analysis but this time without restricting to sites where the ancestral allele could be determined. After grouping sites sharing the same combinations of haplotypes, the major populations were still observed to separate in this analysis (supplementary fig. S9, Supplementary Material online). A PCA based on MNP mutational fractions, that is, those changes for which an intermediate haplotype is not observed, also does not show the definition between continental groups observed for SDMs (supplementary fig. S10, Supplementary Material online).

**Fig. 4.**
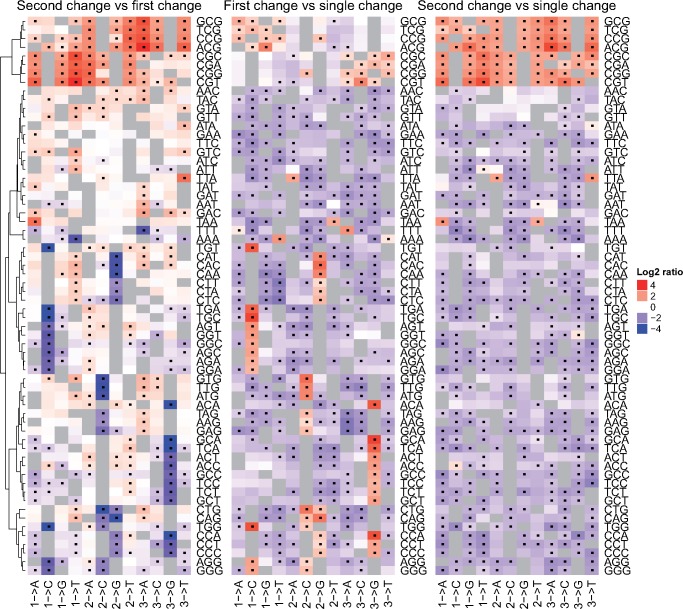
—The relative mutational fractions of different forms of first and second changes among SDMs. (Left) The ratios of the mutation fractions of the first and second changes by triplet context in the 1000 genomes cohort. Each cell corresponds to a particular change defined by the original triplet (labeled on the right of the plot) and the observed change (labeled at the bottom). The numbers in the bottom row indicate which base in the triplet is polymorphic followed by the mutation that occurred, that is, 1 -> A in the bottom GGG row indicates the respective cell corresponds to GGG -> AGG changes. A dot in the cell indicates that the frequency of the corresponding change is significantly different between the first and second changes of SDMs (Bonferroni corrected chi-square *P* < 0.05, see Materials and Methods for more details). (Middle and right) The ratio of the frequencies of the first (middle) and second changes (right) in SDMs versus the observed frequencies of the same change at SNPs. In all three heatmaps, red cells indicate the change is enriched among the first named change, and blue that it is enriched among the second named change.

This improved separation observed in the SDMs analysis is driven by the fact that the first and second changes in SDMs show distinct biases between populations, providing a greater resolution of population differences. This is illustrated by the fact a PCA of the differences in mutational fractions between the first and second change in SDMs also effectively separates the major population groups (supplementary fig. S11, Supplementary Material online). If the first and second change in SDMs showed the same biases between populations, then continental groups should not still separate in this analysis.

Consequently, SDMs show different mutational spectra across populations that are distinct and more pronounced than those among SNPs and MNPs, and which can effectively separate major population groups.

### CpG Deamination Is Not Sufficient to Explain SDM Variation

Intriguingly, the results in supplementary figure S11, Supplementary Material online, imply that the first and second mutations in SDMs are not driven by the same processes. To investigate this further, we characterized the types of mutations observed as the first and second change in SDMs classified again by base change and triplet context. To ensure that batch effects and sequencing artifacts did not confound this analysis, we replicated it across two independent data sets. The global set of 2,504 genomes sequenced by the 1000 Genomes Consortium used above, as well as a novel data set of 1,358 Scottish genomes from the Lothian Birth Cohorts 1921 and 1936 (Deary et al. 2012; Taylor et al. 2018), whole genome sequenced at a mean depth of 36×.

Comparison of the frequency with which particular changes occur as the first and second mutation in an SDM indicates that each shows biases for different types of change. As shown in figure 4, a clear role of CpG dynamics in shaping SDMs is observed. Methylated cytosines immediately followed by a guanine (i.e., CpG sites) are known to be particularly prone to deaminate to a thymine, with mutation rates at these sites up to 18 times higher than at other dinucleotides (Kong et al. 2012). As shown in figure 4, the first mutation in SDMs is more likely to create a new CpG site, with the second change more likely to lead to the loss of one. This suggests that a dominant factor underlying SDMs is an initial mutation that creates a new CpG site, which subsequently mutates. This signature is observed across both the 1000 genomes and Lothian Birth Cohorts (supplementary fig. S12, Supplementary Material online).

**Fig. 5.**
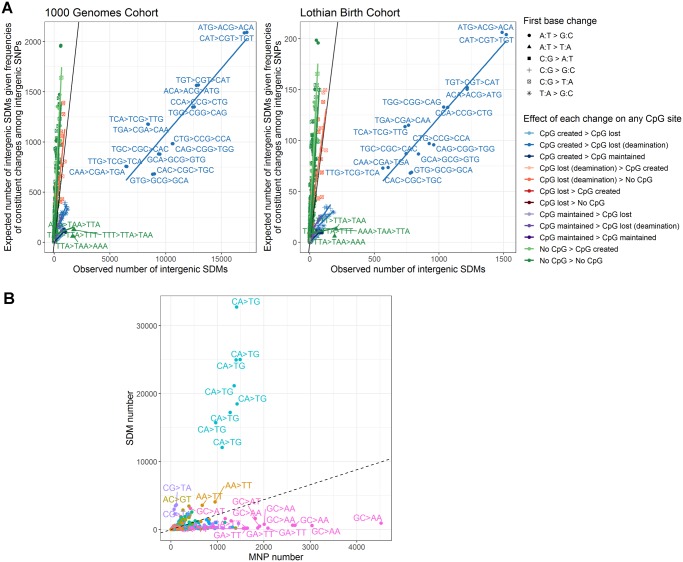
—The observed number of intergenic SDMs against the number expected given the frequencies with which the constituent changes occur among SNPs and MNPs. (*A*) SDMs versus SNPs. SDMs are broken down by the original base change and the impact on any CpG sites of each constituent change (impact of first change -> impact of second change). The line of parity is shown in black. Together, these three factors (the expected number of SDMs from background SNP rates, the first base change and impact of the changes on CpG sites) explain the majority of the variation in SDM frequencies (McFadden’s pseudo-*R*^2^: 0.84 [Lothian Birth Cohort], 0.85 [1000 Genomes Cohort]). (*B*) The number of SDMs versus number of MNPs showing the same ancestral and derived triplets in the 1000 genomes cohort. Points are colored by the dinucleotide change between the ancestral and derived haplotypes and outlier points labeled.

This raises the question, to what extent do SDMs simply reflect the known mutational biases of SNPs (supplementary fig. S13, Supplementary Material online)? To explore this, we determined the expected number of each SDM in the genome given the observed frequencies of occurrence of its constituent changes among SNPs (see Materials and Methods for more details). Moderate correlations were observed between these values (fig. 5*A*, negative binomial regression McFadden’s psuedo-*R*^2^: 0.57 [Lothian Birth Cohort], 0.61 [1000 Genomes Cohort]), but substantial outliers were observed, where the frequencies of occurrence of SDMs in the genome differ markedly from what would be expected given the rates of changes among SNPs. In particular, the SDMs involving CA → CG → TG changes (and their complement TG → CG → CA) occur over an order of magnitude more frequently than expected given the frequency of occurrence of the constituent changes among SNPs (fig. 5*A*). Notably, they also occur over an order of magnitude more often than other changes that involve the creation and subsequent deamination of CpG sites. Whereas 10,633 intergenic CAG → CGG → TGG changes were observed in the 1000 genomes population there are only 1,038 intergenic CTG → CGG → TGG changes, despite both changes leading to a similar creation and loss of CpG sites, and both ancestral triplets occurring at the same frequency in the genome (due to being the reverse complement of one another). This enrichment of these changes is maintained at extended sequence contexts (supplementary fig. S14, Supplementary Material online). This implies that a distinct process where an initial A:T → G:C change is favored has led to the comparatively high number of these changes, and CpG dynamics alone cannot explain the elevated occurrence of these specific SDMs relative to other SDMs involving a final CpG to TpG change. This is further illustrated in supplementary figure S15, Supplementary Material online. Modeling the interaction between these two factors (original base change and the impact of changes on CpG sites) using regression analysis confirms that only where a CpG is created by an initial A → G, and not for example by a T → G change, is the rate of SDMs so high (supplementary fig. S15, Supplementary Material online).

Comparison of the number of SDMs to the number of MNPs sharing the same ancestral and derived triplets in the 1000 genomes cohort further highlights the distinct mutational biases of SDMs and in particular the bias toward the specific enrichment for CA → CG → TG changes (fig. 5*B*). Whereas, as previously observed, MNPs are enriched with GA → TT and GC → AA changes, thought to be a result of error prone replication by polymerase zeta (Harris and Nielsen 2014), SDMs are distinct due to this bias toward CA → CG → TG mutations.

Further mutational biases specific to SDMs are also observed. For example, TTA to TTT polymorphisms are more common as the second change in an SDM than the first, and SDMs containing two consecutive A:T→ T:A changes are more common than expected given the frequency of occurrence of the same changes among SNPs (figs. 4 and 5*A*). Consequently, although the turnover of CpG sites drives the creation of a large proportion of SDMs, further processes appear to be contributing to different biases not only between SDMs and SNPs/MNPs but also between the first and second changes of neighboring polymorphisms.

### The Sequence of Changes Observed Depends on Their Ancestral State

This relationship between the first mutation in SDMs and their frequency of occurrence in the genome raises the question as to whether the second mutation depends to some extent on the first. To explore this, we examined SDMs where initial mutations have created the same intermediate nucleotide triplet. If the two changes that comprise an SDM are independent, then the subsequent mutations at these intermediate triplets should occur in similar numbers irrespective of the ancestral triplet from which they derived.

**Fig. 6.**
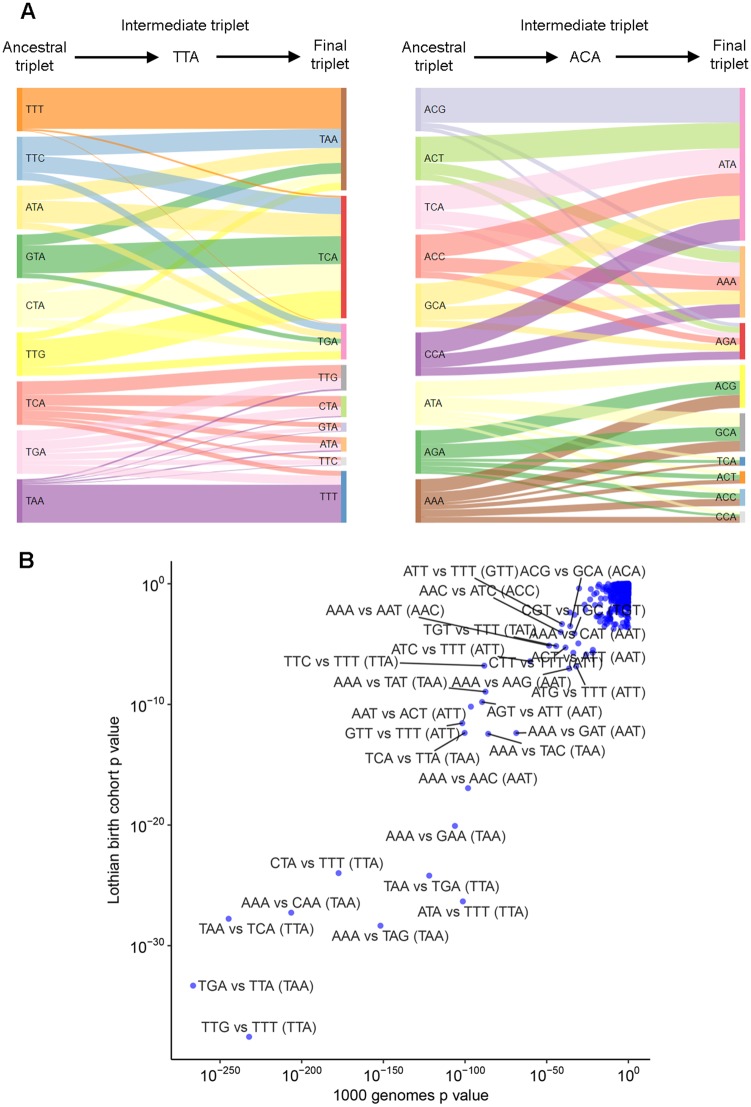
—The form of the second change is related to the ancestral state of SDMs. (*A*) Sankey plots representing the changes observed among intergenic SDMs sharing the same intermediate state. The width of each bar represents the proportion of SDMs sharing the corresponding ancestral state that ended at the indicated final triplet. (*B*) *P* values indicating pairs of ancestral states where intergenic SDMs show different biases for final triplets despite passing through the same intermediate state (indicated in brackets). Chi-square test *P* values for both the 1000 genomes and Lothian Birth Cohorts are shown.

The dominant feature underlying downstream changes at many of these intermediate triplets was the biased deamination of CpG sites. SDMs passing through an intermediate triplet containing a CpG site are dominated by subsequent CpG to TpG changes irrespective of the original ancestral nucleotides. We therefore focused on intermediate triplets that contain no CpG sites. As shown in figure 6*A*, mutations at these intermediate triplets are not independent of the initial change. For example, 94% of SDMs passing through an intermediate TTA triplet go on to become TAA if the original ancestral codon was TTT. This number is though only 17% if the original ancestral codon was TTG (fig. 6*A* and table 1). This link between the form of the first change on the observed fraction of downstream changes was shown to be maintained in expanded sequence contexts (one and two bases either side of each SDM, see supplementary table 1, Supplementary Material online). To minimize the potential impact of batch effects and sequencing artifacts, we again sought replication for this observation in the independent Lothian Birth Cohort collection of Scottish genomes and this difference was found to be highly significant in both data sets (fig. 6*B*).

**Table 1:** Counts and Proportions (by Ancestral Triplet) of SDMs Passing through the Intermediate Triplet TTA

Ancestral Triplet	Intermediate Triplet	Derived Triplet	Intergenic SDMs	Intergenic Proportion by Start Triplet	Intronic SDMs	Intronic Proportion by Start Triplet	Coding SNP Count (First Change)	Coding SNP Count (Second Change)
ATA	TTA	TAA	38	0.34	32	0.42	314	68
ATA	TTA	TCA	56	0.50	36	0.47	314	558
ATA	TTA	TGA	17	0.15	9	0.12	314	91
CTA	TTA	TAA	36	0.21	20	0.19	1,207	68
CTA	TTA	TCA	104	0.61	69	0.64	1,207	558
CTA	TTA	TGA	30	0.18	18	0.17	1,207	91
GTA	TTA	TAA	21	0.25	19	0.30	403	68
GTA	TTA	TCA	54	0.64	34	0.53	403	558
GTA	TTA	TGA	9	0.11	11	0.17	403	91
TTC	TTA	TAA	62	0.44	55	0.49	656	68
TTC	TTA	TCA	54	0.38	43	0.38	656	558
TTC	TTA	TGA	25	0.18	15	0.13	656	91
TTG	TTA	TAA	44	0.17	38	0.20	1,865	68
TTG	TTA	TCA	162	0.64	121	0.64	1,865	558
TTG	TTA	TGA	49	0.19	31	0.16	1,865	91
TTT	TTA	TAA	1,817	0.94	1,696	0.95	257	68
TTT	TTA	TCA	81	0.04	55	0.03	257	558
TTT	TTA	TGA	28	0.01	27	0.02	257	91
TAA	TTA	ATA	61	0.03	52	0.03	15	168
TAA	TTA	CTA	46	0.02	44	0.03	15	606
TAA	TTA	GTA	27	0.01	19	0.01	15	244
TAA	TTA	TTC	23	0.01	18	0.01	15	182
TAA	TTA	TTG	76	0.04	47	0.03	15	1,046
TAA	TTA	TTT	1,611	0.87	1,489	0.89	15	209
TCA	TTA	ATA	80	0.15	55	0.14	1,503	168
TCA	TTA	CTA	130	0.25	90	0.23	1,503	606
TCA	TTA	GTA	58	0.11	43	0.11	1,503	244
TCA	TTA	TTC	35	0.07	34	0.09	1,503	182
TCA	TTA	TTG	160	0.31	127	0.33	1,503	1,046
TCA	TTA	TTT	60	0.11	39	0.10	1,503	209
TGA	TTA	ATA	32	0.14	11	0.06	24	168
TGA	TTA	CTA	47	0.21	29	0.17	24	606
TGA	TTA	GTA	19	0.09	16	0.09	24	244
TGA	TTA	TTC	25	0.11	18	0.10	24	182
TGA	TTA	TTG	54	0.24	43	0.25	24	1,046
TGA	TTA	TTT	45	0.20	55	0.32	24	209

This phenomenon is not exclusively restricted to intermediate triplets containing just adenines and thymines. For example, ACA intermediate triplets are more likely to be linked to an ATA final triplet if the ancestral triplet was CCA than if it was ACG (fig. 6*A* and *B*). There appear therefore to be constraints on the second change in SDMs dependent on their original ancestral state.

### Coding SDMs Have Favored the Creation of Single Codon Amino Acids

**Fig. 7.**
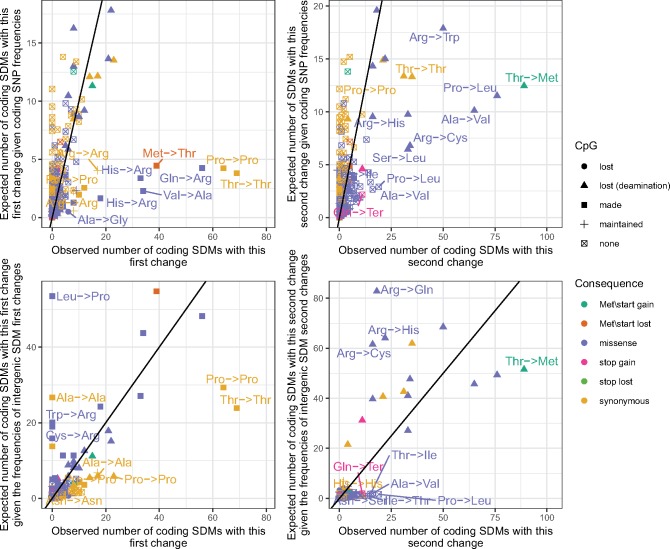
—The observed and expected number of coding SDMs in the 1000 genomes cohort. The *x* axes in the left and right columns correspond to the observed number of first and second changes in coding SDMs, respectively. Each point represents a distinct single base change between two codons and points are colored by the impact of the change on the corresponding protein. The impact of the change on any CpG sites is also indicated by the shape of the point. The *y* axis in the top row corresponds to the number of the changes expected given their frequency among coding SNPs. The *y* axis in the bottom row gives the expected numbers given the frequencies of the changes among the first and second changes of intergenic SDMs after accounting for differences in the frequencies of occurrence of ancestral triplet between regions. Labeled points show a significant difference between the observed and expected numbers after correcting for multiple testing (chi-square *P* < 2.4e^−5^). The line of equality is also indicated.

We next investigated the impact of these mutational biases on coding regions. As shown in figure 7, SDMs have had a distinct impact on the evolution of genes. The particular biases of SDMs means that nine out of ten of the most common coding SDMs involve the previously described CA → CG → TG change. As a result of the layout of the amino acid code, this bias has led to the preferential creation of the single codons that code for methionine and tryptophan (logistic regression of frequencies of changes that create these single codon amino acids to the frequencies of all other changes: *P* = 6.4 × 10^−04^).

Thr_ACA_ → Thr_ACG_ → Met_ATG_ is the most common coding SDM, with Gln_CAG_ → Arg_CGG_ → Trp_TGG_, the fourth most common change (fig. 7 and supplementary fig. S16, Supplementary Material online). The mutational biases of SDMs and organization of the amino acid code have therefore combined to favor the creation of single codon amino acids. The comparatively low number of these amino acids created by SNPs is therefore partially compensated for by the particular mutational biases of SDMs.

To investigate the potential impact of selection in coding regions, we compared the observed number of coding SDMs to that expected given the numbers of the same SDM in intergenic regions, after accounting for differences in the occurrence of the ancestral triplets between regions (see Materials and Methods). Under the assumption that intergenic SDMs are under comparatively little selection, discrepancies between these numbers should indicate which coding SDMs have been favored or disfavored by selection. A noticeable difference to the previous comparison with coding SNPs is that the first change of coding SDMs is depleted with a number of the changes that create a CpG site (fig. 7 and supplementary fig. S17, Supplementary Material online). SDMs where the first mutation is nonsynonymous (missense, stop gained, or lost) are depleted among coding regions, consistent with their removal by selection, with the notable exception of the Thr_ACA_ → Thr_ACG_ → Met_ATG_ pathway through the amino acid code that remains unusually enriched among coding SDMs (observed proportion of coding SDMs matching this change vs. expected proportion given number in intergenic regions and differences in rates of ancestral codon between regions: chi-square *P* = 2.9 × 10^−10^, Bonferroni corrected *P* = 6.58 × 10^−7^). This suggests that this change is favored not only by the mutational biases of SDMs but also by the selection in coding regions.

**Fig. 8.**
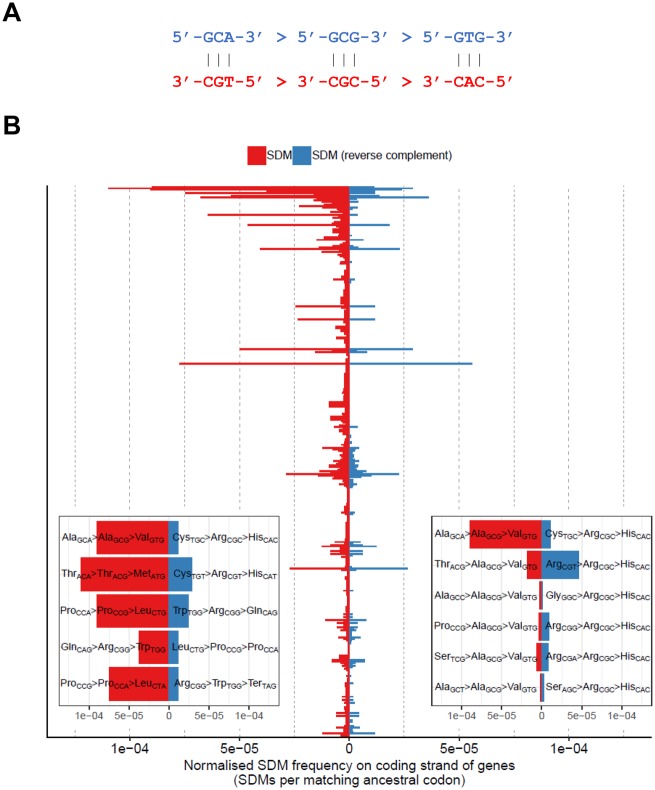
—Asymmetry in the occurrence of changes on the coding and noncoding strand of genes. (*A*) Reverse complement changes are expected to occur at the same rates across the genome. (*B*) The observed count of each SDM change on the coding strand of genes, normalized by the total frequency with which the ancestral codon occurs in coding regions, is shown. Each change is paired with its reverse complement change, with the count of the change displaying a comparative enrichment on the coding strand shown in red. Where both changes are equally common on coding strands, one was randomly chosen to be shown in red. To test for asymmetry in the frequencies of SDM pairs, a 2 × 2 contingency table of (*a*) the count of each SDM in coding regions and (*b*) the count of the matching ancestral codons across all genes was constructed, and differences in the proportion of ancestral codons carrying the respective SDM were tested using the Fishers exact test. SDM pairs are ranked by *P* value (smallest at the top). The five most significant pairs (*P* < 5 × 10^−5^) are shown in the bottom-left inset and the six SDMs involving a final Ala_GCG_ -> Val_GTG_ change are shown in the bottom right.

To explore the mutational profiles of coding SDMs further, we compared the normalized occurrence with which SDMs occur on the coding and noncoding strands of genes. Taking the SDM shown in figure 8*A* as an example, as a change on one strand must also effect the other strand, then the null hypothesis is that in the absence of selection and processes such as transcription coupled repair the numbers of these two complementary changes (GCA > GCG > GTG and TGC > CGC > CAC) should be similar. In this analysis, we compared if though there was an impact of whether a gene was present on the blue or red strand. If the frequencies of these changes are independent of whether or not they occur on the coding strand then the two changes should still occur at the same rates when accounting for the background occurrence of the respective ancestral codon on the coding strand of genes. This is what we see for most changes, that is, an SDM and its reverse complement are found at approximately equal normalized occurrences on the coding strand of genes (fig. 8*B*). However, a subset of SDMs show imbalances in the normalized occurrence with which they and their reverse complement occur on the coding strand, including both the Thr_ACA_ → Thr_ACG_ → Met_ATG_ and Gln_CAG_ → Arg_CGG_ → Trp_TGG_ changes. Of the five most significant SDMs in this analysis, only one does not involve a CA → CG → TG change (Pro_CCG_ → Pro_CCA_ → Leu_CTA_). The reverse complement of this change is Arg_CGG_ → Trp_TGG_ → Stop_TAG_ suggesting that this change may be comparatively infrequent on the coding strand due to its leading to the introduction of a deleterious stop codon.

We conclude that although particular changes are favored by SDM mutational biases, selection has preferentially removed changes passing through certain codons, leaving changes that create single codon amino acids comparatively unaffected. Together, this has led to different impacts on coding regions of SDMs and SNPs.

### Evidence for Epistatic Selection at Neighboring Coding Polymorphisms

We finally investigated whether there is evidence of epistatic selection acting across the polymorphisms that make up coding SDMs, that is, whether the selective pressure acting on a nucleotide change depends on neighboring changes. The amino acid code is thought to have been optimized so that physically similar amino acids have been brought together at neighboring positions (Koonin and Novozhilov 2009). This ensures that the negative effect of a single mutation is minimized. By extension though, this suggests that two successive missense mutations in a codon are potentially more deleterious than one, despite the net change being one amino acid change in both cases, with the strength of selection acting on the first mutation modulated by the allele present at the neighboring base.

To explore this hypothesis, we focused on SDMs where the initial mutation led to a missense change and characterized whether the strength of selection acting upon this initial change depends upon subsequent neighboring mutations in the same codon. If a subsequent change in the same codon is synonymous then the original amino acid change is unaffected and the null hypothesis is that the strength of selection on the codon should be in line with that among coding SNPs with the same change as the original missense mutation. However, if the second change is a further nonsynonymous change and the amino acid code is optimized so that two subsequent missense changes in the same codon are more deleterious than just one, such changes should be relatively depleted in the genome despite the fact there is still only one amino acid change. We used multiple linear regression to control for potential confounders (the occurrence of the first change among coding SNPs indicating the expected occurrence of these changes, the number of different codons that encode the final amino acid and the impact on any CpG site of the second change). Supplementary figure S18, Supplementary Material online, shows that SDMs made up of two successive missense changes are less frequently observed in the genome when compared with missense changes followed by a subsequent synonymous change (*P* = 0.0013, false discovery rate = 0.0088). The number of missense–synonymous SDMs in the genome is on average 37% higher than missense–missense SDMs after accounting for the number of coding SNPs showing the same change as the original missense mutation, CpG mutability, and codon frequency. When also accounting for the state of the intermediate triplet, this difference remains significant (*P* = 0.00015, false discovery rate = 0.010).

Although single codon amino acids appear to have been generally favored by SDMs, having accounted for this effect, the creation of a methionine codon following an original missense change in fact occurs particularly infrequently relative to synonymous changes (*P* = 1.4 × 10^−6^, false discovery rate = 1.3 × 10^−5^). Consequently, the impact of missense changes on fitness appears to depend on the form of subsequent neighboring mutations, and epistatic selection between neighboring coding polymorphisms has helped shape the number of SDMs in the human genome.

## Discussion

Although the changes that make up SDMs have been most often categorized as individual SNPs, we have shown they appear to be driven by distinct mutational processes. Although the creation and subsequent deamination of CpG sites underlies a large proportion of SDMs, we show, we believe for the first time, that CA → CG → TG changes are substantially overrepresented relative to other changes that involve the gain and deamination of a CpG site. This suggests a distinct process is driving this bias for these specific changes. The creation of new G:C base pairs is often attributed to biased gene conversion that favors weak (A:T) to strong (G:C) basepair changes. However, previous studies have found little evidence of a strong effect of biased gene conversion on genome-wide mutational profiles (Do et al. 2015; Harris and Pritchard 2017), and, for example, T:A → G:C changes are not similarly enriched among the first change of SDMs, suggesting that a distinct process is potentially contributing to the elevated rate of these polymorphisms.

To define the SDMs in this study, we relied on Ensembl ancestral allele calls but any misidentification of ancestral alleles could potentially confound the analyses. For example, the enriched CA → CG → TG changes could potentially be attributed to two independent CpG deamination events on different strands if the inferred ancestral genome was incorrect and the CG haplotype was in fact the true ancestral state. This would mean these SDMs were in fact two neighboring SNPs. To minimize this possibility, we restricted the analyses to sites with high-confidence calls that meant that the ancestral allele had to be observed across multiple sequences in a six-way alignment of primate genomes. Most commonly, the same ancestral allele was observed across all five other sequences in the alignment. Despite this, the unusually high frequency of CpG deamination events could potentially lead to sites where all the primate genomes carry the same change on the same strand (i.e., all carry the CG → CA mutation and not the CG → TG mutation) (Hernandez et al. 2007). However, a specific enrichment for SDMs involving the gain and then loss of a CpG site is observed, irrespective of whether the first change can be attributed to a potentially misannotated CpG deamination event. For example, the observed enrichment of GG → CG mutations among the first change in SDMs cannot be attributed to the known high rates of CpG deamination even if the ancestral allele calls were incorrect. So although it is not possible to exclude the possibility of some level of ancestral allele misidentification in this analysis, the gain and loss of CpG sites, in agreement with the ancestral allele calls, provides a parsimonious explanation for this set of changes.

As with SNPs, SDM mutational profiles appear to differ between human groups, but, unlike SNPs and MNPs, SDM rates can more clearly define populations. In contrast to SNP mutational profiles, the SDM profiles of the Americas populations distinguishes them from other continental groups, in part driven by a depletion of CG → TG → TA changes among these individuals. The larger spectrum of changes among SDMs, and the fact that the changes in SDM are not independent, and do not simply reflect underlying SNP rates, likely provides the greater resolution in defining populations. Although genotyping and switch errors would impact the ability to call SDMs accurately, previous analyses have suggested that the accuracy of the 1000 genomes cohort is high and the use of the independent and high coverage (>30×) Lothian Birth Cohort to validate key findings suggests that errors due to low sequencing coverage in 1000 genomes samples are not driving the observations in this study. Our examination of switch errors in 26 randomly selected individuals through read based phasing suggests switch errors are relatively infrequent at these neighboring bases. Likewise, SDMs exhibit distinct mutational biases to MNPs suggesting they are not simply inappropriately annotated MNPs. The first change of most SDMs appears to have occurred prior to the migration out of Africa. All else being equal a second mutation is more likely to occur on an older, more common haplotype but the constraints on defining these changes may also make identifying recent, rare SDMs more difficult.

Not all common SDMs are associated with CpG sites. In particular, changes at AT-rich triplets show population differences and enrichment among African populations. The second change in AT-rich SDMs also often appears dependent on the original ancestral sequence. One potential explanation for this is a role for homologous recombinational repair among these SDMs. Recombination based repair mechanisms transfer nucleotide sequence information between chromosome copies, and as a result between ancestral and derived haplotypes. The second changes in these SDMs may therefore reflect errors in this repair process, potentially arising due to the pre-existing mismatch between chromosomal copies. Alternatively, constraints on the sequence composition of regions may lead to biases toward those maintaining local nucleotide composition. Recent work has highlighted that as many tests of adaptive evolution, such as the branch-site test, assume base substitutions occur independently, then violations of this assumption can lead to erroneous signals of positive selection (Venkat et al. 2018). MNMs have been shown to drive a lot of false positive signals but this potentially also extends to SDMs.

An intriguing consequence of the bias for the creation and subsequent loss of CpG sites among SDMs is the fact that this favors the creation of methionine and tryptophan codons due to the specific arrangement of the amino acid code. As both codons contain a TG dinucleotide, they are readily created by the preference for CA → CG → TG changes. This therefore partly offsets the fact that these amino acids are more rarely created by single mutations due to being only encoded by a single codon. Among coding SDMs, the creation of new methionine codons is the most common of all double mutations, even when accounting for background mutation rates. However, it should be noted that as SDMs are relatively rare, the overwhelming majority of new methionine codons are still created by SNPs.

Nonadditive, that is, epistatic, genetic interactions have been proposed to underlie a range of phenomenon such as the missing heritability of phenotypes, but detecting such interactions in humans has proven difficult (Wei et al. 2014). Previous studies have suggested that the strength of selection acting upon a coding variant may depend on the alleles carried at other variants nearby. For example, Lappalainen et al. (2011) showed that putatively functional coding variants are less often observed on more highly expressed regulatory haplotypes. Given the previous observations that the amino acid code appears optimized so that the more deleterious amino acid changes cannot result from single base changes, we investigated whether SDMs may be a further example of epistatic selection in the human genome. When accounting for various factors, an original nonsynonymous change is less often than expected followed by a nonsynonymous change in the same codon. This suggests that the strength of selection acting upon the original missense mutation is modulated by changes next to it, despite the net effect still being a single amino acid change. However, an assumption made to varying degrees by these studies is that missense changes can be grouped, and that their impact on fitness is broadly similar. Even larger sequencing cohorts, such as those being generated as part of the UK Biobank (Sudlow et al. 2015), would help refine this analysis and the epistatic selection acting upon individual types of intermediate missense changes.

Consequently, human mutation profiles appear more complex than previously thought, with neighboring polymorphisms driven by distinct mutational processes. These SDMs are under unusually strong selective pressure and have played an important and distinct role in shaping human protein evolution.

## Supplementary Material

Supplementary data are available at *Genome Biology and Evolution* online.

## Supplementary Material

Supplementary Data
