# Supplementary figures and images for: Linked Mutations at Adjacent Nucleotides Have Shaped Human Population Differentiation and Protein Evolution
Source: Genome Biol Evol. 2019 Jan 23;11(3):759–75. doi: 10.1093/gbe/evz014 (PMC6424222; doi:10.1093/gbe/evz014)

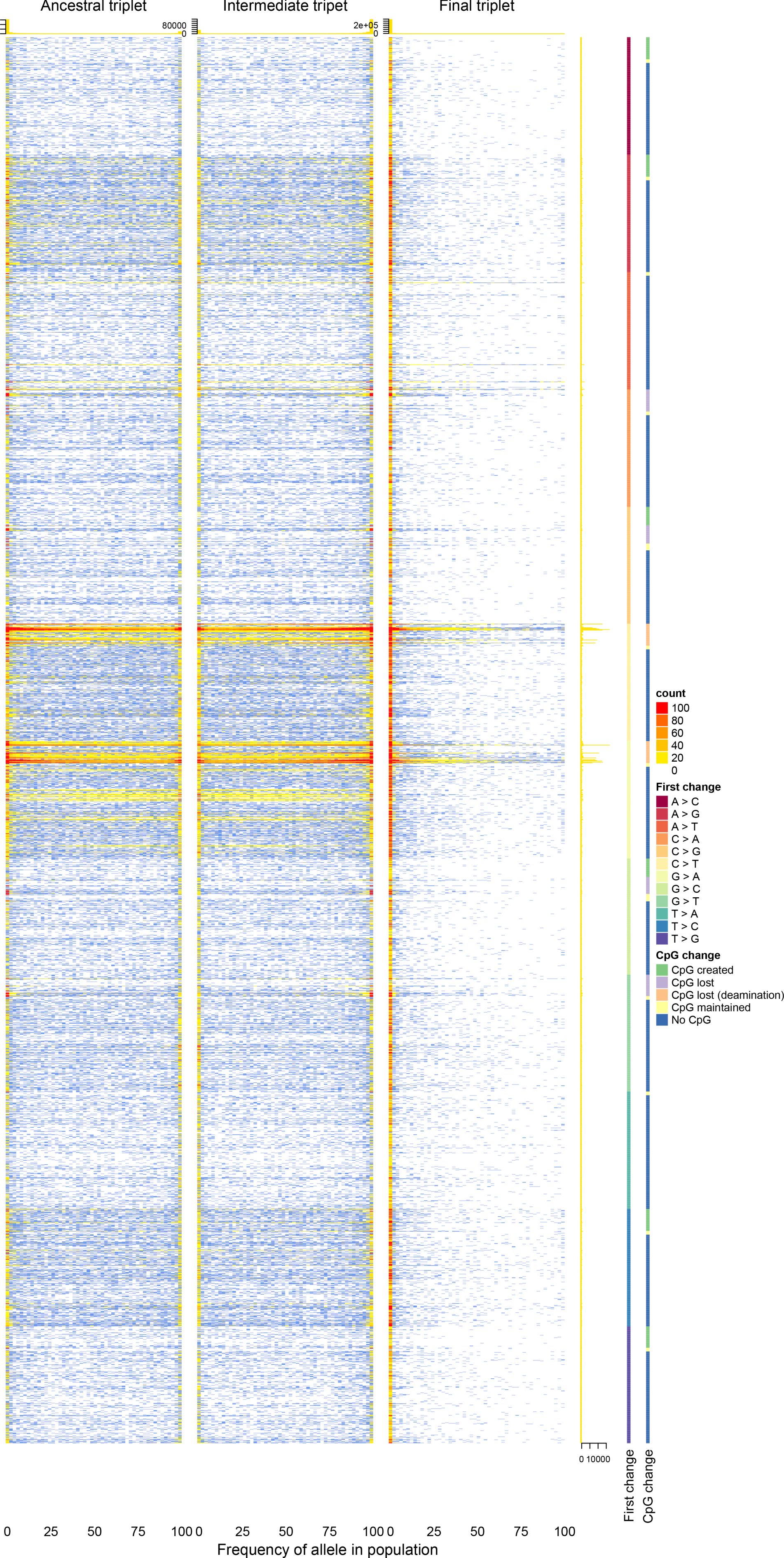

Supplement: Supplementary Data [file evz014_supp.zip › FigureS8.pdf]
